# PD41 Quality Of Diagnostic Test Accuracy Systematic Reviews In Neglected Diseases Registered In PROSPERO: Meta-Epidemiological Review Of Methodological Compliance

**DOI:** 10.1017/S0266462324003040

**Published:** 2025-01-07

**Authors:** Denis Satoshi Komoda, Marilia Mastrocolla de Almeida Cardoso, Brígida Dias Fernandes

## Abstract

**Introduction:**

Diagnostic test accuracy (DTA) systematic reviews (SR) provide acknowledged challenges for health technology assessment (HTA) due to insufficiency of trials and a paucity of methodological frameworks (compared with interventional SRs). Additionally, research in neglected tropical diseases (NTDs) is scarce. Assessing the methodological compliance of a SR protocol in this context is of utmost importance for developing robust HTA in NTD.

**Methods:**

A search strategy was conducted in PROSPERO in November 2023 to identify protocols of SRs on diagnostic test accuracy using the following terms: “diagnostic accuracy test”, “diagnostic test”, and “diagnostic accuracy”. Deduplication was performed using Excel’s “remove duplicates” functionality. Eligible studies were SRs with predicted meta-analyses on DTA in any NTD (as per the World Health Organization list). in vitro studies, those with non-human populations, and methodological studies were ineligible. The variables of interest included registry characteristics (author, year, country), protocol status, and methodological characteristics (pre-defined statistical analyses, sensitivity analyses, and heterogeneity analyses). The results were presented using descriptive statistics and narrative analysis of methodological issues.

**Results:**

From 7,931 registries, 106 protocols were selected that anticipated conducting a meta-analysis of DTA in the context of NTDs. The number of registry entries has grown steadily from one protocol in 2012 to 17 in 2023. Twenty-three NTDs were identified, the three most common being dengue (n=23; 22%), leishmaniasis (n=18; 17%), and syphilis (n=12; 11%). Only 14 protocols were reported as published. Regarding quality of reporting and methods, half (n=54) of the reports explicitly stated a meta-analytical approach in their title. Only 16 mentioned a sensitivity analysis and 32 did not mention an analysis of heterogeneity.

**Conclusions:**

The meager number of protocols reported as published could mean a lack of updates or publications, or both. The absence of essential methodological descriptions in protocol titles and body text is worrisome and may ultimately be reflected in the number of publications. Further analysis should assess the specifics of methodological quality based on well-established methodological frameworks for quality of reporting and methods.

